# Pharmacological Modulation and (Patho)Physiological Roles of TRPM4 Channel—Part 1: Modulation of TRPM4

**DOI:** 10.3390/ph15010081

**Published:** 2022-01-10

**Authors:** Zsigmond Máté Kovács, Csaba Dienes, Tamás Hézső, János Almássy, János Magyar, Tamás Bányász, Péter P. Nánási, Balázs Horváth, Norbert Szentandrássy

**Affiliations:** 1 Department of Physiology, Faculty of Medicine, University of Debrecen, 4032 Debrecen, Hungary; kovacs.zsigmond@med.unideb.hu (Z.M.K.); dienes.csaba@med.unideb.hu (C.D.); hezso.tamas@med.unideb.hu (T.H.); almassy.janos@med.unideb.hu (J.A.); magyar.janos@med.unideb.hu (J.M.); banyasz.tamas@med.unideb.hu (T.B.); nanasi.peter@med.unideb.hu (P.P.N.); horvath.balazs@med.unideb.hu (B.H.); 2 Doctoral School of Molecular Medicine, University of Debrecen, 4032 Debrecen, Hungary; 3 Division of Sport Physiology, Department of Physiology, Faculty of Medicine, University of Debrecen, 4032 Debrecen, Hungary; 4 Department of Dental Physiology and Pharmacology, Faculty of Dentistry, University of Debrecen, 4032 Debrecen, Hungary; 5 Faculty of Pharmacy, University of Debrecen, 4032 Debrecen, Hungary; 6 Department of Basic Medical Sciences, Faculty of Dentistry, University of Debrecen, 4032 Debrecen, Hungary

**Keywords:** TRPM4, SUR1, CBA, flufenamic acid, 9-phenanthrol, siRNA, antibody, TRPM4 activator, TRPM4 inhibitor

## Abstract

Transient receptor potential melastatin 4 is a unique member of the TRPM protein family and, similarly to TRPM5, is Ca^2+^-sensitive and permeable to monovalent but not divalent cations. It is widely expressed in many organs and is involved in several functions by regulating the membrane potential and Ca^2+^ homeostasis in both excitable and non-excitable cells. This part of the review discusses the pharmacological modulation of TRPM4 by listing, comparing, and describing both endogenous and exogenous activators and inhibitors of the ion channel. Moreover, other strategies used to study TRPM4 functions are listed and described. These strategies include siRNA-mediated silencing of TRPM4, dominant-negative TRPM4 variants, and anti-TRPM4 antibodies. TRPM4 is receiving more and more attention and is likely to be the topic of research in the future.

## 1. Introduction

Transient receptor potential (TRP) channels were discovered in Drosophila when the structure of a protein fulfilling a role in phototransduction was described in 1989 [1]. The 28 members of the TRP family are divided into six subfamilies based on the sequence homology among the members. These include TRP canonical (TRPC1-7), TRP vanilloid (TRPV1-6), TRP melastatin (TRPM1-8), TRP ankyrin 1 (TRPA1), TRP mucolipin (TRPML1-3), and TRP polycystin (TRPP2, TRPP3, and TRPP5) [2]. The subfamily of transient receptor potential melastatin (TRPM) consists of eight members (TRPM1-8), forming four pairs based on the similarity in their sequence [3]. A pair is made of TRPM4 and TRPM5, which are unique, as they are only permeable to monovalent but not divalent cations [4,5]. Ion substitution experiments revealed a selectivity sequence of Na^+^ > K^+^ > Cs^+^ > Li^+^ for TRPM4 [6,7]. TRPM4 is activated by intracellular Ca^2+^ [8,9].

The role of a protein can be examined both in vitro and in vivo. Among these, one must mention the pharmacological intervention of the given protein using compounds modifying its function [4] as well as genetic approaches such as knock-down (KD) or knock-out (KO) [10] and overexpression of the protein [11]. Studying the function in vivo is definitely more complicated, as whole animal models need to be used. These models complicate both the pharmacological and genetic approaches, as the compound must be added in a way that provides an effective concentration [12]. Furthermore, KD and/or overexpression might need to be performed in an organ-/tissue-specific manner [13,14]. The in vivo approach, however, can be translated to humans more easily than in vitro methods. Evaluating the function of a protein in vitro is a good starting point to understand its function on the level of the whole organism. When modulating (either activating or inhibiting) the channel activity, the selectivity of the applied compound is very important to avoid misinterpreting the results.

This part of the review summarizes the current state of knowledge on the modulation of TRPM4 by endogenous compounds, drugs, and other approaches. Part 2 of the review describes the physiological and pathophysiological roles of TRPM4 in various tissues.

## 2. Activation of TRPM4

**Figure 1:**
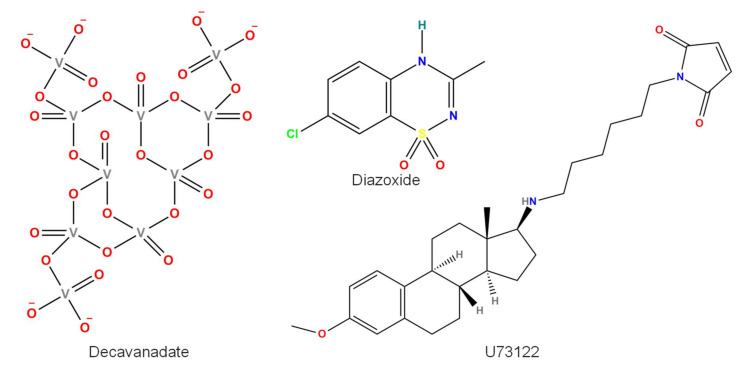
Chemical structure of compounds directly activating TRPM4 or the sulfonylurea receptor 1 (SUR1)-TRPM4 co-assembled channel (diazoxide). U73122 can also activate TRPM4 in an indirect manner. All structures were created by ChemDrawPro 12.0 software.

**Figure 2:**
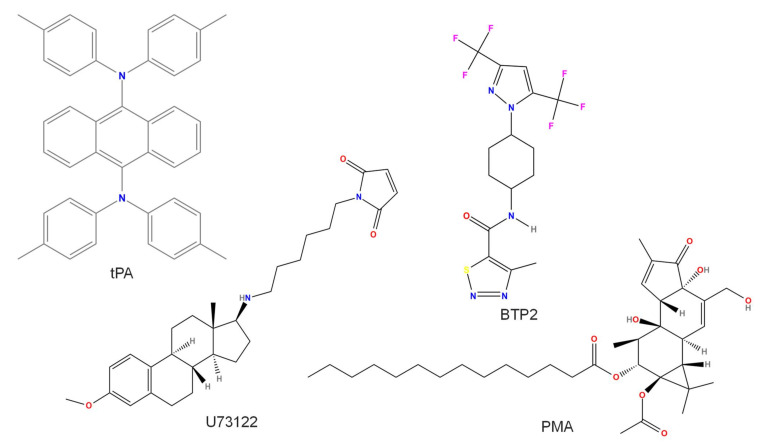
Chemical structure of compounds activating TRPM4 indirectly. U73122 also activates TRPM4 directly. All structures were created by ChemDrawPro 12.0 software.

Both endogenous and exogenous compounds can increase the TRPM4 current. Some compounds act directly on the channel protein (Figure 1); others have other targets and indirectly increase the TRPM4 current (Figure 2). The first one to mention is intracellular Ca^2+^ itself [7]. In addition, phosphatidylinositol 4,5-bisphosphate (PIP2) (and its related compounds) [9,15,16] and calmodulin [11,17] must be mentioned as important endogenous compounds activating TRPM4 from the intracellular space. Other compounds, such as decavanadate [18], 3,5-bis(trifluoromethyl)pyrazole derivative (BTP2) (also known as YM-58483) [19], H_2_O_2_ [20], tissue plasminogen activator (tPA) [21], U73122 (an inhibitor of phospholipase C) [22], and the adenosine triphosphate-dependent K^+^ (K_ATP_) channel activator diazoxide [17], need to be mentioned. Last but not least, phosphorylation induced by protein kinase C (PKC) also increases the TRPM4 current [11].

### 2.1. Ca^2+^

As TRPM4 is an ion channel activated by both voltage and Ca^2+^, a certain minimum concentration of intracellular Ca^2+^ must be present for its activation. Both the minimum concentration and the value of the half effective activator concentration (EC_50_) depend on several factors, most of which are related to experimental conditions. These include the mode of recording (cell-attached patch, perforated or whole-cell configuration, inside-out patch)—most likely due to the presence or loss of intracellular regulatory compounds—the value of the membrane potential, and the studied preparation (overexpressed or native channels). In addition, it has been well established that even with a given stable free Ca^2+^ concentration applied to the intracellular side, the TRPM4 current is subjected to rapid desensitization in the range of 1–2 min after the establishment of the recording configuration [16,18]. On the contrary, the TRPM4 current of rat dental follicle stem cells, the HCT116 colorectal cancer cell line, and TRPM4-expressing human embryonic kidney (HEK) cells showed no desensitization despite the tight-seal whole-cell recording condition [23,24,25]. The reason for this discrepancy is not clear. Gonzales et al. suggested that the proper amount and type of intracellular Ca^2+^ buffering are required to avoid TRPM4 current desensitization, at least in smooth muscle cells [26]. Interestingly, based on the study of Gonzales et al., desensitization could be avoided only in the HCT116 colorectal cancer cell line recording when 10 mM EDTA (a slower Ca^2+^ buffer than EGTA) was applied. The other two studies used 10 mM BAPTA as a Ca^2+^ buffer, so it is not clear why desensitization was not detected. Nevertheless, due to the abovementioned rapid desensitization, the determination of EC_50_ also depends on the time of determination. Indeed, the EC_50_ values during the steady state reached after current desensitization were 5 times higher (110 vs. 524 µM) in excised patches of Chinese hamster ovary (CHO) [16] and 30 times higher (4.4 vs. 140 µM) in HEK cells [18], both overexpressing human TRPM4. As a result of all the abovementioned factors, the Ca^2+^ concentration required for TRPM4 activation is reported in a broad range. For instance, the minimum Ca^2+^ concentration for channel activation detected in the inside-out configuration in native sinoatrial and ventricular cardiomyocytes or native CHO cells was between 0.1 and 1 µM [9,27,28]. EC_50_ values of free intracellular Ca^2+^ were reported in the range of 0.4–80 and 10–1000 µM in the case of whole-cell configuration and inside-out configuration, respectively. The values of the Hill coefficient, if determined, also varied in a broad range from 0.6 to 3.7 in the case of inside-out recordings and even slightly higher (5–6) in whole-cell measurements [7]. The reason for the broad range for values of both the EC_50_ and Hill coefficients might be due to the different experimental conditions (cell type used, different solutions, and voltage).

EC_50_ values were also drastically dependent on the presence of PIP2. Application of 10 µM diC8-PIP2 (the water-soluble and nonmetabolizable form of PIP2) in a pipette increased Ca^2+^ sensitivity by 100 times (from 134 to 1.3 µM) [15] in HEK cells and 5 times (from 524 to 123 µM) in CHO cells [16]. Surprisingly, the Hill coefficient did not change after the application of PIP2 in HEK cells (0.9 and 1.0) but doubled in CHO cells (1.5 vs. 2.9). The effect of PIP2 is also discussed in detail later.

The binding site for Ca^2+^ was also described in detail recently. Four amino acids (Glu828, Gln831, Asn865, and Asp868) located close to the S3 transmembrane helix in hsTRPM4 coordinate a Ca^2+^ ion [29,30,31]. As TRPM4 functions as a homo- or heterotetramer [32], the abovementioned Hill coefficients exceeding a value of 1 might be due to the binding of one Ca^2+^ ion to each unit of the tetramer.

Recently, the K^+^ channel tetramerization domain 5 protein was indicated as a novel TRPM4-interacting protein, which enhances the Ca^2+^ sensitivity of TRPM4 and thereby promotes the cell migration and contractility observed in breast cancer [33]. Similarly, Rho-associated protein kinase also activates TRPM4 by increasing its Ca^2+^ sensitivity and is involved in the regulation of myogenic tone [34].

### 2.2. Phosphatidylinositol 4,5-Bisphosphate (PIP2)

TRPM4 activation is very effectively facilitated by the presence of intracellular PIP2 (IUPAC name: [(1R,2S,3R,4R,5S,6R)-2,3,5-trihydroxy-4-[[2-[(5Z,8Z,11Z,14Z)-icosa-5,8,11,14-tetraenoyl]oxy-3-octadecanoyloxypropoxy]-oxidophosphoryl]oxy-6-phosphonatooxycyclohexyl] phosphate) [16]. The molecule activates the channels by increasing their sensitivity to Ca^2+^ and shifting their activating voltage to more negative potentials [15]. PIP2 had an EC_50_ of 5.1–5.8 µM and a Hill coefficient of 2.1–2.6 to activate TRPM4 (i.e., recover the channel from desensitization), but other related compounds can also modulate TRPM4 [15,16]. The number of phosphate groups in the molecule is important, as doubly phosphorylated PIs such as PI(3,4)P2 and PI(3,5)P2 were equally effective, and the monophosphorylated form PI(4)P had no effect, while the triple-phosphorylated PI(3,4,5)P3 was more effective than PIP2 itself [16]. On the contrary, the triple-phosphorylated PI(3,4,5)P3 was less effective than PIP2 itself, and the double-phosphorylated forms showed similar activity to PI(3,4,5)P3 [15]. In addition, among the monophosphorylated forms, PI(5)P had similar effectivity to the triple-phosphorylated PI(3,4,5)P3, but PI(3)P and PI(4)P were ineffective [15]. This suggests that phosphorylation on position 5 makes the molecule more effective in inducing recovery from the desensitization of TRPM4. The two previously mentioned studies were conducted on expressed TRPM4 channels, but 10 µM PIP2 tripled the open probability of native TRPM4 channels of freshly isolated murine sinoatrial node cells, even with 1 µM free intracellular Ca^2+^ concentration [9]. Another approach for the evaluation of the role of PIP2 is to stimulate a phospholipase C (PLC)-coupled signaling pathway or to apply a PLC inhibitor (5 µM U73122) to prevent the degradation of PIP2. This latter approach greatly enhanced Ca^2+^ sensitivity in the case of TRPM4 channels on murine renal primary cilia [35]. Another strategy to prove the importance of PIP2 for TRPM4 is to reduce the PIP2 concentration by either applying the PIP2 scavenger poly-l-lysine [16], delaying the regeneration of PIP2 by applying wortmannin, the inhibitor of PI-4-kinase, or using inositol polyphosphate 5-phosphatases [15]. Cai et al. showed that cholesterol activates TRPM4 via a PIP2-dependent mechanism in murine cortical collecting duct cells by increasing its Ca^2+^ sensitivity [36].

The binding site for PIP2 was suggested to be located on the first putative pleckstrin homology domain located on the C-terminus of the protein [15]. Alternatively, the pre-S1 region of the N-terminus was proposed to host the PIP2 binding site [37]. Arg755 and Arg767 of hTRPM4 were shown to be crucial in the interaction with PIP2 [31]. Recently, another site located on the C-terminus was indicated for PIP2 binding [38].

PIP2 was shown to interfere with other ion channels of the TRP family, including the tonic inhibition of TRPV1 [39]. Other TRP channels such as TRPM5 [40], TRPM7 [41], TRPM8 [42], and TRPV5 [43,44] are activated by PIP2. Other non-TRP ion channels are also activated by PIP2. TREK1 and KCNQ1 are activated by PIP2 by shifting their activation curves to more negative potentials [45]. P/Q-type Ca^2+^ channels are influenced by 20 µM PIP2 in two opposing ways: stabilization of channel activity and voltage-dependent inhibition [46]. Cloned and native N-type Ca^2+^ channels are also modulated by PIP2, as 10 µM reversed the rundown of their current [47]. Furthermore, PIP2 influences other ion channels, such as voltage-gated, inwardly rectifying, and Ca^2+^-activated potassium channels [48]. In addition, voltage-gated Ca^+^ channels, TRP channels, epithelial Na^+^ channels, P2X receptors, TMEM16A channels, and cyclic nucleotide-gated channels are on the long list of ion channels affected by PIP2 [48]. As the concentration of PIP2 required to stimulate TRPM4 is in a similar range to that which affects other ion channels, it seems that PIP2 application in a native cell is not selective enough to study the function of TRPM4.

### 2.3. Calmodulin

TRPM4 is also modulated by the presence of calmodulin [11,17]. In HEK cells transiently expressing human TRPM4, the effect of calmodulin was studied by either adding calmodulin to the intracellular side of the channels, which doubled the open probability, or by using dominant-negative calmodulin mutants. The mutation or deletion of the three calmodulin binding sites located in a short range of the C-terminus enhanced TRPM4 activation by Ca^2+^ and delayed its desensitization. In contrast, mutation or deletion of the two calmodulin binding sites at the N-terminus did not affect TRPM4 function [11]. Woo et al. reported doubled calmodulin binding affinity upon coexpression of sulfonylurea receptor 1 (SUR1) with TRPM4 channels compared with that of TRPM4 alone [17]. Two additional binding sites located at the N- and C-termini were identified as capable of binding calmodulin, S100A1, and PIP2 [38].

### 2.4. Decavanadate

TRPM4 activity is enhanced by the intracellular application of the six negative charges contained within decavanadate (DV, IUPAC name: [5,13-bis[(dioxido(oxo)vanadio)oxy]-1,3,5,7,9,11,13-heptaoxo-2,4,6,8,10,12,14,15,16-nonaoxa-1lambda5,3lambda5,5lambda5,7lambda5,9lambda5,11lambda5,13lambda5-heptavanadatricyclo[9.3.1.13,7]hexadecan-9-yl]oxy-dioxido-oxovanadium, Figure 1), the decamer form of vanadate [18]. When applied to the intracellular side of a patch made from HEK cells containing transiently expressed human TRPM4 channels, 10 µM DV induced a fast and fully reversible increase in the inward current but hardly changed outward currents, indicating a strong effect on the voltage-dependent gating of TRPM4. The DV-induced increase in inward currents was concentration-dependent, having an EC_50_ of 1.9 µM and a Hill coefficient of 1.8 [18]. The maximum current increase was 2.5 times compared with that in the absence of DV. In addition, adenosine triphosphate (ATP)-induced TRPM4 inhibition was increased in the presence of 5 µM DV by 10 times, but its voltage dependence was unaltered. This makes the competitive binding of ATP and DV unlikely. Deletion of the highly positively charged 1136RARDKR sequence from the C-terminus but not the 332RDRIRR sequence from the N-terminus resulted in the loss of the DV effect, suggesting the importance of the C-terminal site in mediating DV action [18]. The abovementioned Hill coefficient value (nearly 2) suggests two binding sites with positive cooperativity. Although the N-terminal site was not suggested to be important to DV action, Winkler et al. reported two functional DV binding sites [49]. One of these was the previously mentioned site on the C-terminus containing six positively charged amino acids, and another one is located in the interface between TRPM homology regions 1/2 and 3 from the adjacent subunit of the TRPM4 tetramer, where three positively charged arginine residues are found [49].

In addition to TRPM4, DV acts on P2X-type purinergic receptors [50]. P2X7 receptors expressed in HEK cells were the most sensitive, but decavanadate also blocked P2X2 and P2X4 receptors. DV at a concentration of 10 µM induced an almost complete blockade of inward currents of P2X7 receptors in a rapid and reversible manner [50]. In permeabilized rat pancreatic acinar cells, DV antagonizes the binding of inositol 1,4,5-trisphosphate (IP3) to its receptors, with an EC_50_ of 5 µM, and 20 µM DV was required for complete inhibition [51]. Inhibition of Ca^2+^-ATPase of the sarcoplasmic reticulum required at least 40 µM DV [52]. These data indicate that the application of DV in cellular preparations is not suitable for studying TRPM4.

### 2.5. BTP2 or YM-58483

TRPM4 activity was increased by the compound called 3,5-bis(trifluoromethyl)pyrazole derivative (BTP2), also known as YM-58483 (IUPAC name: *N*-[4–3,5-bis(trifluromethyl)pyrazol-1-yl]-4-methyl-1,2,3-thiadiazole-5-carboxamide, Figure 2) [19]. It must be noted that BTP2 is often used as an inhibitor of the mainly ORAI channel-mediated Ca^2+^-release-activated current [53]. In HEK cells overexpressing TRPM4, BTP2 was only effective in the presence of some Ca^2+^, and preincubation with 10 µM BTP2 resulted in much higher TRPM4 currents. BTP2 increased the native TRPM4 current of Jurkat cells in a concentration-dependent manner with an EC_50_ of 8 nM [19]. Regarding the specificity of BTP2, it must be noted that in Jurkat cells, the Ca^2+^-release-activated current is inhibited in a range from 0.5 to 4 µM, and a similar concentration was needed to inhibit TRPC3 and TRPC5 channels [54], so BTP2, if applied at low enough concentrations, can be a useful tool to investigate TRPM4 in native cells too.

### 2.6. H_2_O_2_

The desensitization of TRPM4 channels overexpressed in HEK cells can be eliminated in a dose-dependent manner by H_2_O_2_ [20]. This effect was observed both in whole-cell and inside-out recording configurations and can be detected starting from 50 µM. HeLa cells were less sensitive to H_2_O_2_-induced cell death. The TRPM4 mutation of Cys1093Ala reduced the sensitivity to H_2_O_2_ but not to PIP2. Therefore, Simon et al. suggested that redox modifications of the Cys1093 residue could increase calmodulin binding, leading to reduced desensitization of TRPM4 [20]. Pretreatment of the cardiomyocyte cell line H9c2 with 200 µM H_2_O_2_ induced cell death. Silencing of TRPM4 and pretreatment with 9-phenanthrol, a TRPM4 inhibitor, prevented H_2_O_2_-induced cell death [55]. H_2_O_2_-induced TRPM4 stimulation was described in human umbilical vein endothelial cells [56]. In addition, the expression of TRPM4 was increased by 200 µM H_2_O_2_ in those cells [57]. Importantly, in mpkCCDc14 cells, TRPM4 activity in a mouse cortical collecting duct principal cell line was not influenced by even 500 µM H_2_O_2_, but 100 µM H_2_O_2_ pretreatment for 24 h significantly reduced TRPM4 expression on the apical membrane, suggesting that H_2_O_2_ inhibits its trafficking [58]. At a higher concentration (10 mM), H_2_O_2_ activated TRPM2 channels of both primary lens epithelial cells and the cell line HLE-B3 [59]. Other TRP channels are also sensitive to redox changes: H_2_O_2_ activated TRPM2, TRPC5, TRPV1, and TRPA1 too [60].

### 2.7. Tissue Plasminogen Activator (tPA)

In murine brain endothelial cells, tPA (Figure 2) opened SUR1-TRPM4 channels in a plasmin-, PAR1-, TRPC3-, and Ca^2+^-dependent manner [21]. Recombinant tPA (20 μg/mL) induced both macroscopic and single-channel SUR1-TRPM4 currents. De novo expression of SUR1-TRPM4 channels in response to NF-κB activation was also observed [21]. Other targets of tPA include toll-like receptors in CSF-1 macrophages, which are selectively inhibited by 12 nM tPA [61], and *N*-methyl-d-aspartic acid (NMDA) receptors purified from the cerebral cortex of C57BL/6mice, which mediate the effect of 12–100 nM tPA [62]. Calculating the tPA concentration used by Gerzanich et al. yields approximately 286 nM, which is at least 3 times higher than that in the other two previously mentioned studies. Thus, tPA is definitely not a good choice to study TRPM4 function in cellular preparations.

### 2.8. U73122

U73122 (IUPAC name: 1-[6-[[(8R,9S,13S,14S,17S)-3-methoxy-13-methyl-6,7,8,9,11,12,14,15,16,17-decahydrocyclopenta[a]phenanthren-17-yl]amino]hexyl]pyrrole-2,5-dione, Figure 1 and Figure 2) is an inhibitor of phospholipase C, and therefore, it is often used to study the effect of PIP2 on the activity of various ion channels, including TRPM4 (see above). U73122 influenced TRPM4 via direct interaction: activation of TRPM4 by 5 µM U73122 was independent of PIP2 and Ca^2+^ [22]. Interestingly, TRPM5 channels were not influenced, but TRPM3 channels were blocked by 5 µM U73122, indicating the high specificity of U73122 within the TRPM channel family [22]. U73343, a compound used as a negative control (not inhibiting phospholipase C), induced almost the same effect as U73122 on I_KAch_ of mouse atrial myocytes: full inhibition by 10 µM and a half inhibitory concentration (IC_50_) value of 160 nM, slightly higher than that of U73122 (120 nM) [63]. Similarly, in HEK cells, both U73122 and U73343 potently blocked expressed BK and Kir3 channels, most likely by acting on their homologous domain within long C-terminal ends [64]. G-protein-coupled inward rectifier K^+^ channels are also significantly blocked by 1 µM U73122 and even more potently blocked by 1 µM U73343 [65]. In addition, 10 µM U73122 activated Ca^2+^ release from intracellular stores of mouse pancreatic acinar cells in a phospholipase C–independent manner [66]. Studies that use the compound as a PLC inhibitor usually apply 5–10 µM U73122 [15,35]. It seems that U73122 is not selective enough and, therefore, at least in native cells, definitely cannot be used as a TRPM4 activator.

### 2.9. Diazoxide

The most well-known action of diazoxide (IUPAC name: 7-chloro-3-methyl-4H-1lambda6,2,4-benzothiadiazine1,1-dioxide, Figure 1) is the activation of K_ATP_ channels, enabling its use in the treatment of hypoglycemia [67]. As mentioned earlier, TRPM4 can form heteromer channels with SUR1, a subunit of K_ATP_ channels [68]. Therefore, it is not surprising that diazoxide influences TRPM4-SUR1 coexpressed channels in COS-7 cells [17]. In these coexpressed channels, 100 µM diazoxide greatly increased the current (approximately by 15 times) but had no effect in cells expressing either TRPM4 or SUR1 alone. Diazoxide at a concentration of 100 µM also strongly activated native TRPM4-SUR1 channels in human brain endothelial cells [21]. As diazoxide non-selectively activates K_ATP_ channels, it stimulates Kir6.2/SUR1 channels in β-cells, Kir6.2/SUR2B and Kir6.1/SUR2B channels of smooth muscle, and, to a small extent, also cardiac Kir6.2/SUR2A channels [69]. As TRPM4 can function without SUR1 and K_ATP_ channels potentially exist in native cells, it is unlikely that diazoxide can be used as an activator of TRPM4 currents, or at least caution must be applied.

### 2.10. PKC-Mediated Phosphorylation

TRPM4 currents are increased by the phosphorylation of TRPM4 channels by PKC. The PKC activator phorbol 12-myristate 13-acetate (PMA, IUPAC name: [(1S,2S,6R,10S,11R,13S,14R,15R)-13-acetyloxy-1,6-dihydroxy-8-(hydroxymethyl)-4,12,12,15-tetramethyl-5-oxo-14-tetracyclo[8.5.0.02,6.011,13]pentadeca-3,8-dienyl] tetradecanoate, Figure 2), applied in a 1 h preincubation at 1 µM in HEK cells transiently expressing human TPRM4, reduced the EC_50_ value for Ca^2+^ from 15 to 4 µM [11]. Moreover, the chance for TRPM4 activation by 1 µM Ca^2+^ greatly increased in the case of PMA pretreatment from 3 of 12 cells to 7 of 10 cells. A similar effect was observed with 10 min pretreatment with 0.5 µM PMA in inside-out TRPM4 current detection (probably by increasing Ca^2+^ sensitivity) in various native cell types, including dedifferentiated ventricular cardiomyocytes [70], freshly dissociated human right atrial cells [4], and ventricular cells isolated from spontaneously hypertensive rats [27]. The sites responsible for the action include the serine amino acids at positions 1145 and 1152, as their mutation to alanine led to the loss of effectivity of PMA [11].

## 3. Inhibition of TRPM4

**Figure 3:**
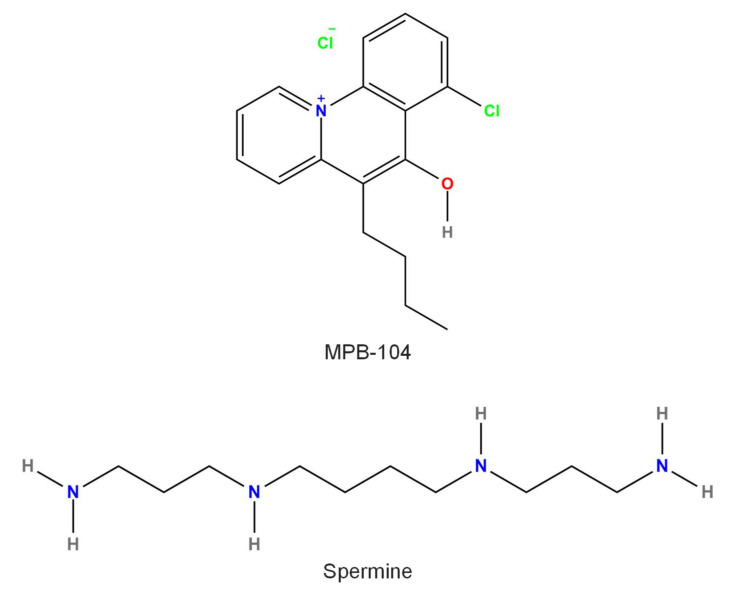
Chemical structure of compounds causing TRPM4 inhibition via a putative binding site. All structures were created by ChemDrawPro 12.0 software.

**Figure 4:**
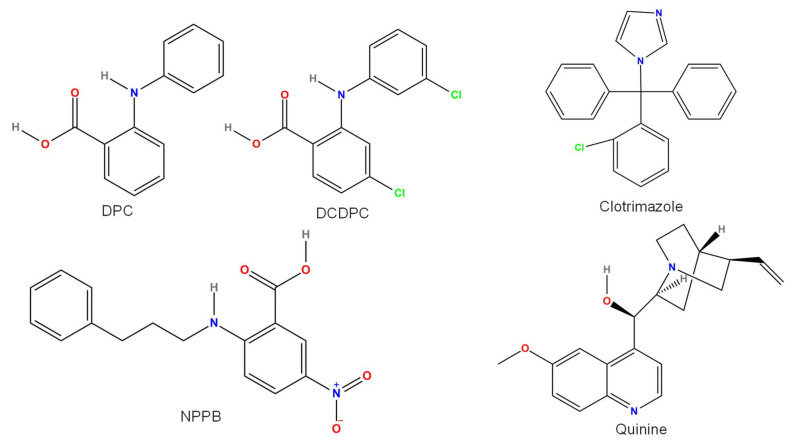
Chemical structure of compounds causing TRPM4 inhibition in a nonspecific manner. All structures were created by ChemDrawPro 12.0 software.

**Figure 5:**
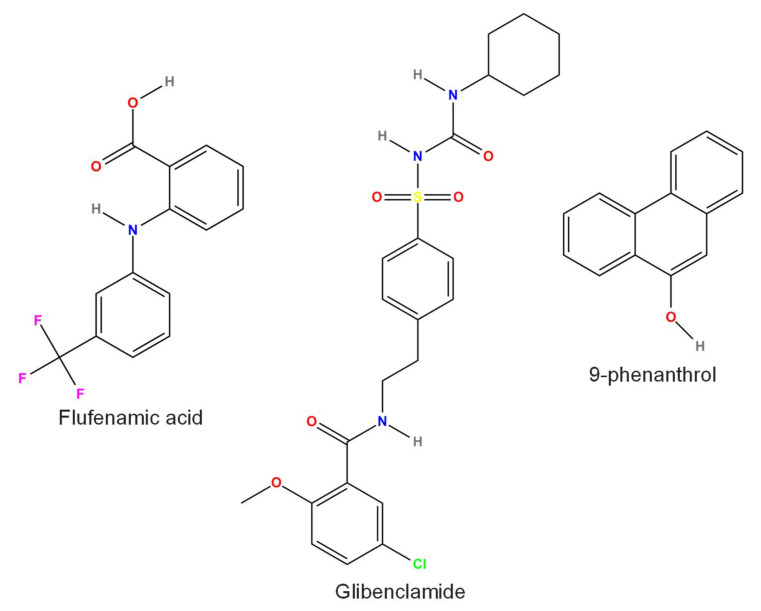
Chemical structure of compounds widely used for TRPM4 inhibition. All structures were created by ChemDrawPro 12.0 software.

**Figure 6:**
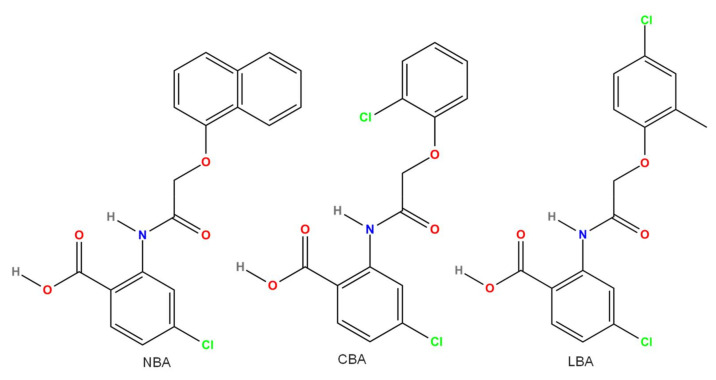
Chemical structure of the newest TRPM4 inhibitors. All structures were created by ChemDrawPro 12.0 software.

Several compounds block TRPM4 current (Figure 3, Figure 4, Figure 5 and Figure 6). Some of these are endogenous molecules, such as adenosine triphosphate (ATP) (and other related molecules) [71], nitric oxide (NO) [72], and spermine [71]. Others are exogenous compounds, including quinine [73], MPB-104 [74], the nonsteroid anti-inflammatory compound flufenamic acid (FFA) [75], the antidiabetic drug glibenclamide [4], the antimycotic drug clotrimazole [76], chloride channel blockers (such as diphenylamine-2-carboxylic acid (DPC), 3′,5-dichlorodiphenylamine-2-carboxylic acid (DCDPC), and 5-nitro-2-(3-phenylpropylamino)benzoic acid (NPPB)) [77], and 9-phenanthrol [77]. The selectivity of these compounds is, in many cases, rather weak, necessitating the search for and testing of newer and newer compounds. Such recently developed drugs are 4-chloro-2-[[2-(2-chlorophenoxy)acetyl]amino]benzoic acid (CBA), 4-chloro-2-(1-naphthyloxyacetamido)benzoic acid (NBA), and 4-chloro-2-(2-(4-chloro-2-methylphenoxy)propanamido) benzoic acid (LBA) [78,79]. Moreover, especially in in vivo studies, either specific antibodies M4P, M4M, and M4M1 were used to block TRPM4 [80,81], or small-interfering RNAs (siRNA) were applied to silence TRPM4 [82]. This latter approach was used in vitro as well [35].

Lastly, some studies have used the approach of using dominant-negative TRPM4 splice variants to study the contribution of the channel to physiological processes.

### 3.1. Adenosine Triphosphate (ATP)

As TRPM4 can form heteromer channels with SUR1, a subunit of K_ATP_ channels, it is not surprising that ATP (IUPAC name: [[(2R,3S,4R,5R)-5-(6-aminopurin-9-yl)-3,4-dihydroxyoxolan-2-yl]methoxy-hydroxyphosphoryl] phosphono hydrogen phosphate) and other related compounds can inhibit TRPM4 [71]. There is, however, a discrepancy between the action of ATP and that of diazoxide (see above), despite the fact that both of these compounds act on K_ATP_ channels. It is not the direction of action (ATP blocks and diazoxide stimulates) but rather the abundance of data reporting ATP-induced TRPM4 inhibition in both the presence and absence of SUR1 in the channel. On the contrary, diazoxide-mediated activation of TRPM4 was only observed when SUR1 was also expressed with TRPM4 (see above).

Another intriguing fact is that MgATP increased TRPM4 currents (or, to be precise, reversed the desensitization of the current), which was not detected with ATP alone [11] or with Na2ATP, MgADP, and MgGTP [16]. These observations make it unlikely that Mg^2+^ itself plays a role, and the most accepted explanation for the MgATP-induced recovery of TRPM4 currents is the ability of MgATP to activate phosphatidylinositol 4-kinase. This kinase is able to regenerate PIP2 [83], the compound that was mentioned above to stimulate TRPM4. It is noteworthy that 2 mM MgATP reversibly inhibited 87% of the total Ca^2+^-activated current in murine renal primary cilia [35], but the authors concluded that it is likely due to the direct inhibition of ATP and the very low level of PIP2 and/or phosphatidylinositol 4-kinase. Another explanation for the increase in TRPM4 currents by MgATP may be the presence of SUR1, as it is proposed to be facilitated by MgATP [84].

As mentioned before, not only ATP itself but also related compounds such as ADP, AMP, and AMP-PNP reduced the TRPM4 current expressed in HEK cells [71]. These compounds induced inhibition with IC_50_ values of 1.7 µM (free ATP), 2.2 µM (ADP), and 19 µM (AMP and AMP-PNP); in addition, adenosine itself blocked TRPM4 too, with a much higher IC_50_ value (630 µM). On the contrary, GTP, UTP, and CTP at concentrations up to 1 mM did not inhibit the current [71]. Sensitivity to ATP was observed not only for the TRPM4 channels expressed in HEK cells but also for TRPM4 channels endogenously present in CHO cells, which were effectively blocked by 100 μM ATP, ADP, and AMP (reduction in the open probability of 90–99%) [28]. Furthermore, in other cells, including murine sinoatrial node cells [9], vomeronasal sensory neurons [85], dedifferentiated rat ventricular cells [70], left ventricular cells of spontaneously hypertensive rats [27], and human right atrial cells [4], ATP reduced the open probability of TRPM4 channels. The binding site of ATP can be at the intersubunit interface between the nucleotide binding domain and ankyrin repeat domain. In more detail, three amino acids—His160, Trp214, and Phe228—can take part in ATP binding, the first one having particular importance [86]. Surprisingly, ATP inhibited both TRPM4 and SUR1-TRPM4 channels with a similar potency, but ADP and AMP did not block SUR1-TRPM4 channels [87].

ATP is a powerful inhibitor of TRPM4, but because it also blocks K_ATP_ channels, it is not used to study the role of TRPM4 channels.

### 3.2. Nitric Oxide (NO)

Nitric oxide inhibited a current possessing similar characteristics to those of TRPM4 in macrovascular endothelial cells, as several NO donor compounds, such as sodium nitroprusside, S-nitroso-*N*-acetylpenicillamine, and 3-morpholinosydnonimine, all reduced the current when applied at 10–30 µM [72]. The inhibition developed slowly and was voltage-independent. Inhibition of NO breakdown by superoxide dismutase led to current reduction, while nitro-l-arginine (NO synthase inhibitor) potentiated the current [72]. Interestingly, NO reduced TRPM4 channels indirectly via IP3R-associated PKG substrate-mediated inhibition of IP3R-dependent Ca^2+^ release in vascular smooth muscle cells [88]. NO (applied as 100 µM sodium nitroprusside) blocked the K_ATP_ channels of pancreatic β-cells [89]. These K_ATP_ channels were activated by 100 µM l-arginine-generated NO in vascular smooth muscle cells [90]. The *N*-methyl-d-aspartate receptor channel was also influenced by NO via S-nitrosylation [91], and cardiac L-type Ca^2+^ channels were likely to be blocked by NO [92]. Large-conductance Ca^2+^-dependent K^+^ channels and other K^+^ channels were also activated by 10 µM NO in canine colonic smooth muscle cells [93]. As NO is a short-lived molecule and has several other ion channels among its targets, it is not used as a TRPM4 inhibitor.

### 3.3. Spermine

TRPM4 can be blocked by spermine (IUPAC name: *N*,*N*′-bis(3-aminopropyl)butane-1,4-diamine, Figure 3), an endogenous polyamine [94]. In HEK cells expressing the murine TRPM4 or TRPM5 (its closest relative) channels, intracellularly applied spermine induced similar inhibition of both channels (IC_50_ values of 35 and 37 µM, respectively) [8]. Human TRPM4 expressed in HEK cells is also sensitive to spermine (IC_50_ of 61 µM), and the negatively charged amino acid residues of E981, D982, D984, E988, and E996 were suggested to be responsible for the binding [71]. Spermine at a concentration of 1 mM reduced not only expressed but also endogenous TRPM4 currents by approximately 60% in CHO cells [28]. Spermine, similarly to other polyamines, is not specific for TRPM4, as inward rectification of the expressed Kir2.1 channel was increased by 20 µM spermine [95]. Among glutamatergic receptors, kainate receptors were potentiated [96], but AMPA receptors were blocked by spermine [97]. At 20 µM, spermine greatly reduced TRPM7 current as well [98]. Spermine, similarly to NO, modifies the function of many other ion channels besides TRPM4; therefore, it is not used as a TRPM4 inhibitor.

### 3.4. Quinine

Quinine (IUPAC name: (R)-[(2S,4S,5R)-5-ethenyl-1-azabicyclo[2.2.2]octan-2-yl]-(6-methoxyquinolin-4-yl)methanol, Figure 4) reversibly blocked human TRPM4 channels overexpressed in HEK cells in a dose- and voltage-dependent manner, with IC_50_ values of approximately 100–500 µM [73]. TRPM5 was even more sensitive, as the IC_50_ of quinine was 50 µM at −50 mV vs. 450 µM for TRPM4. In addition, TRPM7 and small-conductance Ca^2+^-activated K^+^ channels (Kca1.1 and Kca3.1) were also inhibited by 30 µM quinine [99]. Due to the lack of selectivity, quinine is not suitable for examining the role of TRPM4.

### 3.5. MPB-104

The cystic fibrosis transmembrane conductance regulator (CFTR) channel activator MPB-104 (IUPAC name: 5-butyl-7-chloro-6-hydroxybenzo[c]quinolizinium chloride, Figure 3) [100] blocked TRPM4 channels permanently expressed in HEK cells [74]. MPB-104 reduced TRPM4 when applied from the intracellular side during inside-out recording without a significant effect on single-channel conductance. The inhibition was rapid, reversible, voltage-independent, and achieved with IC_50_ values in the range of 10–20 µM. CFTR-dependent ion efflux was activated by 250 µM MPB-104 [74]. The EC_50_ value of MPB-104 on CFTR activation was approximately 2 µM, measured as ion flux in CFTR channels expressed in CHO cells [101]. The suggested binding site for MPB-104 is an ABC signature-like motif in both CFTR and TRPM4; however, the fact that the former channel is activated but the latter one is blocked by the compound suggests a different mechanism of action and still needs to be confirmed. Nevertheless, MPB-104, similarly to the previously mentioned inhibitors, has not been applied as a TRPM4 inhibitor.

### 3.6. Flufenamic Acid (FFA)

The nonsteroid anti-inflammatory compound FFA (IUPAC name: 2-[3-(trifluoromethyl)anilino]benzoic acid, Figure 5) was widely used as a promising TRPM4 blocker, especially in the beginning of TRPM4 research. An excellent review summarized the role of FFA in research by describing the effects of FFA on many ion channels and its effects at various levels (starting from molecular and ending at the whole system) [75]. FFA is not selective, as its IC_50_ for TRPM4 is between 3 and 6 µM [8,27], whereas for expressed GABA-induced or voltage-gated chloride currents, it is between 4 and 20 µM [102,103]. For any other ion channels influenced by FFA, the EC_50_ (or IC_50_) values are at least 20 µM and range as high as 1 mM [75]. These half effective concentrations provide a large enough “therapeutic window” for FFA. As both expressed [20] and native TRPM4 channels [4,104] can be blocked by FFA, it is still used to study the function of TRPM4. This is highlighted by the fact that even after the emergence of 9-phenathrol, a more selective TRPM4 inhibitor, in 2008 (see below), FFA was still used in a few studies [105,106,107], although in most cases, it was compared with 9-phenathrol.

### 3.7. Glibenclamide

As was mentioned previously, TRPM4 can form heteromer channels with SUR1. Glibenclamide (IUPAC name: 5-chloro-*N*-[2-[4-(cyclohexylcarbamoylsulfamoyl)phenyl]ethyl]-2-methoxybenzamide, Figure 5), a known blocker of K_ATP_ channels (also containing a type of SUR), induced a more potent inhibition of TRPM4-SUR1 coexpressed channels than of TRPM4 alone [17]. Intracellularly applied glibenclamide (100 μM) greatly (by 78%) reduced TRPM4 expressed in HEK cells [9]. Even more potent inhibition was observed in HEK cells (10 μM glibenclamide, reduced to 7% of control) [20]. Glibenclamide, applied to the intracellular side at a concentration of 10 μM, reduced the open probability of native TRPM4 to approximately 20–30% of the control in human right atrial cells [4] and in the left ventricular cells of spontaneously hypertensive rats [27]. As low as 300 nM glibenclamide blocked native TRPM4-SUR1 channels in human brain endothelial cells [21], while TRPM4 channels on detrusor smooth muscle cells were less sensitive (100 μM was used) [108]. Glibenclamide reduced both components of the transient outward K^+^ current with an IC_50_ of 50 µM in mouse ventricular myocytes [109]. At 100 μM, glibenclamide inhibited expressed CFTR channels [110] and several Kv channels in human atrial and ventricular myocytes [111]. Therefore, the low selectivity of glibenclamide makes its use unideal for studying TRPM4, especially in tissue containing K_ATP_ channels. Despite this, glibenclamide is still used in many studies to test the function of TRPM4, especially in relation to the nervous system [112].

### 3.8. Clotrimazole

The antimycotic drug clotrimazole (IUPAC name: 1-[(2-chlorophenyl)-diphenylmethyl]imidazole, Figure 4) blocked TRPM4, with an IC_50_ in the range of 1–10 µM [76]. Clotrimazole also reduced the TRPM2 current (IC_50_ of 0.7 µM) in rat cardiac fibroblasts [113] and irreversibly blocked recombinant human TRPM2 expressed in HEK cells at 30 µM [114]. Clotrimazole effectively inhibited intermediate-conductance Ca^2+^-activated K^+^ channels [115] and the slow component of transient outward K^+^ current in mouse ventricular cells [109] with IC_50_ values of approximately 0.15 and 8 µM, respectively. Similarly, 5, 25, and 50 µM clotrimazole rapidly and mostly irreversibly inhibited the L-type Ca^2+^ current of guinea-pig ventricular cardiomyocytes by 16, 59, and 93%, respectively [116]. Clotrimazole reduced not only native L-type Ca^2+^ current but also recombinant human cardiac L-type Ca^2+^ channel α1C subunits stably expressed in HEK cells [117]. These findings make the use of clotrimazole as a TRPM4 inhibitor very unlikely.

### 3.9. DPC, DCDPC, and NPPB

Some aromatic compounds (Figure 4), such as DPC (diphenylamine-2-carboxylic acid, IUPAC name: 2-anilinobenzoic acid), DCDPC (3′,5-Dichlorodiphenylamine-2-carboxylic acid, IUPAC name: 5-nitro-2-(3-phenylpropylamino)benzoic acid), and NPPB (IUPAC name: 5-nitro-2-(3-phenylpropylamino)benzoic acid), are known as blockers of chloride channels. These molecules blocked Ca^2+^-activated channels (being permeable to monovalent cations and usually referred to as NSC_Ca_ before the discovery and characterization of TRPM4). This inhibition has been reported in various tissues, such as rat exocrine pancreatic cells [118], cochlear outer hair cells [119], and murine renal tubular cells [120]. At 10 and 100 µM, DCDPC reduced the open probability of NSC_Ca_ to 43 and 17% of the control in murine renal tubules, respectively [120]. NPPB and DPC at a concentration of 100 µM reduced I(NSC_Ca_) on the basolateral membrane of rat exocrine pancreatic cells (decreased the open probability from 0.5 in the control to 0.2 and 0.04, respectively) [118]. Despite the inhibition of I(NSC_Ca_) by DPC, DCDPC, and NPPB, their chemical structures share little similarity with those of other TRPM4 blockers and are not used at all for TRPM4 inhibition.

### 3.10. 9-Phenanthrol

One of the most widely used TRPM4 inhibitors is 9-phenanthrol (IUPAC name: phenanthren-9-ol, Figure 5). Grand et al. described 9-phenanthrol in 2008 [74], and six years later, a thorough review detailed the effects of 9-phenanthrol on both recombinant and endogenous TRPM4 channels [77]. Guinamard et al. discussed the specificity of the compound and summarized its actions on smooth muscle, on the heart, and on neuronal activity. The contribution of TRPM4 to cell death and angiogenesis was also discussed [77]. The IC_50_ of 9-phenanthrol was in the range of 17–20 µM in both whole-cell and inside-out patch recordings, and its effect was reversible and voltage-independent [74]. Interestingly, a much smaller IC_50_ (1.7 nM) was reported in human adipose-derived stem cells, but the drug was used in pretreatment [121]. 9-Phenanthrol-induced TRPM4 inhibition was reversible, although removal of the inhibition was more difficult than with FFA [122]. 9-Phenanthrol reversibly reduced the duration of the action potential (AP) of murine atrial cells with an IC_50_ of 21 µM [123]. TRPM4 channels in freshly isolated rat cerebral arterial smooth muscle cells were shown to be slightly more sensitive to 9-phenanthrol (IC_50_ was 11 µM) [124]. Even 100 µM 9-phenanthrol had no effect on TRPM5 [74]. On the contrary, 9-phenanthrol inhibited cAMP-dependent protein kinase and myosin light chain kinase of bovine heart with an IC_50_ of 10 µM [125]. Although 10 µM 9-phenanthrol did not alter the voltage-gated Ca^2+^ and K^+^ channels of primary cardiomyocytes, at 100 µM, it induced 47 and 43% reductions in these channels, respectively [122]. In contrast, as low as 3–30 µM 9-phenanthrol significantly reduced several K^+^ currents in native cardiomyocytes [126]. 9-Phenanthrol blocked cardiac sodium channels and dose-dependently inhibited late and peak sodium currents of rabbit ventricular cells with IC_50_ values of 18 and 71 μM, respectively [127]. Endothelial cell KCa3.1 channels were activated by 20 µM 9-phenanthrol, presumably by direct channel activation [128]. 9-Phenanthrol blocked TMEM16A-induced currents in rat arterial smooth muscle myocytes with an IC_50_ of 12 µM and modified channel gating, suggesting that it is not a pore blocker of TMEM16A [129]. Moreover, 9-phenanthrol auto-fluoresces at 340 nm, which, however, at 10 µM, accounted for only 6 nM of the intracellular Ca^2+^ level [105]. At 30 µM, 9-phenanthrol (if applied only from the intracellular side) inhibited human but activated murine TRPM4 channels stably overexpressed in human embryonal kidney (TsA-201) cells [130]. This must be kept in mind when 9-phenathrol is about to be used in mouse, as the compound might diffuse through the cell membrane due to its lipophilic nature. These results highlight the fact that although 9-phenanthrol is a good and potent TRPM4 inhibitor, it has effects on other channels. This must be taken into account upon its application, especially due to its narrow “therapeutic window”.

### 3.11. CBA and Other Related Compounds (LBA and NBA)

Luckily, CBA (IUPAC name: 4-chloro-2-[[2-(2-chlorophenoxy)acetyl]amino]benzoic acid), a more potent drug with good selectivity, was recently developed [78]. CBA is commercially available and evokes a reversible and approximately 15 times stronger TRPM4 current inhibition compared with 9-phenanthrol. Moreover, CBA reduces endogenous TRPM4 currents even more effectively compared with expressed TRPM4 (IC_50_ values of 1.1 and 1.8 µM, respectively). In addition, even at 10 µM, where at least 90% TRPM4 current inhibition was detected, CBA hardly influenced other important channels, such as TRPM5, GABA A receptor α1 subunit, NMDA receptor, or L-type calcium (judged by a reduction in specific antagonist binding only) [78]. The cardiac Kv11.1 (hERG) channel showed only <5% inhibition of dofetilide binding in the presence of 10 µM CBA. On the contrary, in canine left ventricular cells, 10 µM CBA reduced transient outward K^+^ and late Na^+^ currents by 20 and 47%, respectively [131]. Apart from being a TRPM4 blocker, CBA acts as a chemical chaperone that reduces TRPM4 degradation by the endoplasmic reticulum-associated process [78]. The effectivity of CBA was confirmed on the endogenous TRPM4 current of lymph node carcinoma of prostate (LNCaP) cells [132]. It must be noted that in contrast to the results of Ozhathil et al., the inhibition was only partially reversible; additionally, 20–30% of the TRPM4 current remained even in the presence of 10 µM CBA [132]. The reason for this discrepancy cannot be explained by a difference in cells, as both studies used LNCaP cells. In another prostate cancer cell line, human-derived DU145 cells, CBA was even less effective, as it reduced endogenous TRPM4 current by 55 and 65% at 3 and 50 µM, respectively [132]. Even more importantly, CBA affected TRPM4-influenced cellular functions (proliferation and migration) in these DU145 cells only in very high doses (≥25 µM). CBA actions on cancer hallmark functions (cell viability, migration, cell cycle shift, and adhesion) seemed to be independent of TRPM4, as these processes were present in DU145 cells after the KO of TRPM4 [132]. Borgström et al. suggested the long-term treatment and/or the remaining TRPM4 current in the presence of high CBA doses as a potential reason for the TRPM4-independent toxic effects [132]. Indeed, the previously mentioned chemical chaperone action of CBA might be responsible and serve as an explanation.

In addition to CBA, two other compounds were described as potent TRPM4 inhibitors: NBA (IUPAC name: 4-chloro-2-(1-naphthyloxyacetamido)benzoic acid) and LBA (IUPAC name: 4-chloro-2-(2-(4-chloro-2-methylphenoxy)propanamido) benzoic acid) [78]. Compared to CBA, NBA and LBA were even more potent on TRPM4 channels expressed in HEK cells (IC_50_ values were 0.4 and 1.6 µM, respectively). In the case of native TRPM4 channels, NBA was the most potent blocker, with an IC_50_ of 0.13 µM in the HCT116 colorectal cancer cell line [133], while LBA was slightly less potent compared to CBA (IC_50_ values of 1.84 and 1.17 µM, respectively). Regarding their specificity, 10 μM NBA influenced glucagon-like peptide receptor 1, while 10 μM NBA slightly potentiated TRPM5 currents [78]. Although both NBA and LBA seemed to be slightly more potent blockers of endogenous TRPM4 currents in LNCaP cells, interestingly, neither was able to generate complete inhibition [132]. Both compounds exerted practically irreversible inhibition, and LBA was also less potent (IC_50_ of 0.7 μM) in TRPM4 inhibition compared with NBA (IC_50_ of 0.2 μM). At a concentration of 50 μM, NBA and LBA blocked approximately 90 and 85% of the endogenous TRPM4 currents in DU145 cells, but neither compound influenced the proliferation and migration of these cells [132]. HCT116 cells were more sensitive than LNCaP cells, as complete and irreversible TRPM4 inhibition could be achieved by all three drugs [133]. It seems that although CBA, NBA, and LBA are very potent inhibitors of TRPM4, their effect is less pronounced in cells that have endogenous TRPM4 channels than in those with overexpressed ones. Moreover, unlike with 9-phenanthrol, their inhibition did not lead to functional consequences. CBA and NBA were more effective than 9-phenanthrol and glibenclamide in preventing glutamate-induced neuronal cell death in vitro [134]. On the contrary, similar effectivity was reported in neurons, where membrane potential oscillations and the respiratory output were strongly reduced by both 9-phenanthrol (30 µM) and CBA (50 µM) in a comparable manner [135]. It must be noted that CBA was applied at a large concentration, and, as its selectivity is not thoroughly established yet, off-target effects could be involved. A recent study compared CBA, NBA, and 9-phenanthrol; NBA was the best, as it reduced stably overexpressed human and murine TRPM4 channels with similar effectivity (IC_50_ values between 0.1 and 0.2 µM) upon extra- and intracellular application [130]. Surprisingly, CBA was not effective on murine TRPM4 channels, while it blocked human TRPM4 with IC_50_ values of 0.7 and 0.8 µM when applied extra- and intracellularly, respectively. Comparing the cytotoxicity of the three drugs, NBA and CBA possessed similar IC_50_ values (332 and 545 µM, respectively), but 9-phenanthrol was much more toxic (IC_50_ of 20 µM), at least in non-TRPM4 transfected TsA-201 cells [130]. Clearly, more studies are required to determine the use of CBA, NBA, and LBA in the research of TRPM4 functions.

### 3.12. M4P, M4M, and M4M1 Anti-TRPM4 Antibodies

Due to the absence of an ideal (potent, selective, and able to act when applied in vivo) TRPM4 inhibitor, Chen et al. reported another approach for the evaluation of the (patho)physiological role of TRPM4 by the generation of M4P [80]. This antibody binds to a region close to the channel pore and is capable of inhibiting TRPM4 current and downregulating TRPM4 surface expression. Furthermore, M4P can also be used in vivo by intravenous injection. In a rat cerebral ischemia–reperfusion model, M4P attenuated cerebral injury on both histological and functional levels by reducing the increase in TRPM4 expression [80]. M4P exerted an anti-oncotic effect on various cell types within the brain, including neurons, astrocytes, and vascular endothelial cells [136]. M4P was selective for TRPM4, as it did not influence either TRPM5 [80] or TRPM2 and TRPM7 channels [136]. Recently, Low et al. developed two mouse monoclonal antibodies (M4M and M4M1) targeting an extracellular epitope of human TRPM4 [81]. Interestingly, these antibodies reduced ion currents mediated by expressed human TRPM4 channels but failed to be effective in vivo in rats, highlighting the specificity of TRPM4 inhibition by these truly selective antibodies.

### 3.13. siRNA Approach

Due to the absence of a highly specific pharmacological TRPM4 inhibitor, other approaches are used to study the function of TRPM4. Small interfering RNAs (siRNA) are short (19–30 base pairs long) double-stranded RNA sequences and have been used for a long time to investigate protein functions by reducing their expression, which is achieved by the posttranscriptional silencing action of siRNA [137,138]. TRPM4 silencing was used both in vitro [139,140] and in vivo [141,142]. The application of specific TRPM4 siRNA is compared, in many cases, with the effect of pharmacological inhibition and with the effect of a negative control siRNA (a scrambled sequence that cannot bind to any known sequence). The effectivity of silencing by siRNA is usually tested at least at the mRNA and, in many cases, also at the protein level. In the case of TRPM4 silencing, this effectivity was nearly complete in cells (about 80% of the cells in the treated cerebral arteries) in which the uptake of siRNA occurred (in the remaining 20% of cells, siRNA treatment did not change the TRPM4 expression) [139]. Similarly, the reduction in TRPM4 protein was approximately 70% in rat posterior cerebral artery segments [143]. In other studies where cell cultures were treated with siRNA, the quantity of TRPM4 at both mRNA and protein levels was 20% of the nontreated cells (and also cells treated with the scrambled sequence) [140]. TRPM4 mRNA was reduced by 50–75% with siRNA treatment in two prostate cancer cell lines (DU145 and LNCaP) [144], and 75% reduction in mRNA and approximately 50% reduction in TRPM4 protein was achieved in human umbilical vein endothelial cells (HUVEC) [145] and leukemia cell lines with the MLL gene rearrangement [146]. In addition, siRNA not only reduced TRPM4 expression but also effectively prevented the expression of TRPM4 upon in vivo application in permanent middle cerebral artery [141] and bilateral common carotid arteries occlusion rat models [142]. None of the previous studies reported nonspecific actions of the siRNA treatment; no other proteins (and/or their mRNA), including other TRP channels such as TRPC3, TRPC6 [139], and TRPM7 [140], were influenced by the siRNA treatment targeting TRPM4. This was the case for all of the players of the store-operated calcium entry (ORAI1, ORAI2, ORAI3, STIM1, and STIM2) as well [144]. Table 1 lists studies using siRNA for TRPM4 silencing.

### 3.14. Dominant-Negative Splice Variants

Another approach to study the role of TRPM4 is to reduce its function by expressing a splice variant, which blocks the wild-type variant. Two variants are available in reports. One was first described in HEK cells, in which a single amino acid modification (D984A) led to nonconducting channels [148]. Stable overexpression of the D984A dominant-negative TRPM4 splice variant in the colorectal cancer cell line HCT116 resulted in the complete inhibition of the current without a reduction in TRPM4 protein expression [24]. Similarly, no current could be detected upon overexpression of the D984A dominant-negative TRPM4 splice variant in a human prostate cancer cell line (DU145 cells) [132].

The second mutant lacks the first 177 amino acids in the N-terminus (ΔN-TRPM4) and hardly conducts any current in Jurkat cells [147]. Using ΔN-TRPM4, the TRPM4 channels are present on the cell membrane but fail to conduct ions. Similar to the effect of siRNA-mediated TRPM4 silencing, ΔN-TRPM4 expression reduced lipopolysaccharide-induced endothelial cell death [140]. TRPM4-mediated current was greatly reduced in the rat insulinoma cell line INS-1 expressing the ΔN-TRPM4 variant, and glucose- or arginine-vasopressin-induced insulin secretion was also lower [25]. Moreover, in the same cell line, the ΔN-TRPM4 variant abolished calcium signals and insulin secretion, while another TRPM4 construct lacking the last 160 amino acids in the C-terminal region (ΔC-TRPM4, not suppressing the channel function) failed to influence calcium signals and insulin secretion [149]. Table 2 shows studies applying TRPM4 dominant-negative variants.

## 4. Conclusions

Although TRPM4 was first described at the beginning of the 21st century, reports about a Ca^2+^-activated nonspecific cationic current in various tissues were published long before that. Since then, a tremendous amount of knowledge has been accumulated, but there are still problems to be solved. For instance, the currently used inhibitors are still not selective enough; therefore, new approaches are being developed to circumvent selectivity issues. Therefore, currently, some use the silencing of TRPM4 expression and/or function to elucidate the role of TRPM4. These molecular biological approaches seem to be more specific at the moment, but their application is more complicated compared to using activator or inhibitor compounds. Nevertheless, TRPM4 has become a promising therapeutic target in central nervous system injuries and might also be involved in treatments of other conditions in the future.

## Figures and Tables

**Table 1:** Functional evaluation of TRPM4 using siRNA-mediated silencing.

Studied Preparation	Results	Conclusion	Reference
Rat cerebral arteries	80% reduction in TRPM4 mRNA	TRPM4 channels are regulated by Ca^2+^ release from IP3 receptor	[139]
Rat posterior cerebral artery segments	70% reduction in TRPM4 mRNA	Epithelial sodium channels and TRPM4 interact and contribute to pressure-induced vasoconstriction	[143]
Prostate cancer cell lines	50–75% reduction in TRPM4 mRNA	TRPM4 contributes to cancer cell migration	[144]
Human umbilical vein endothelial cells (HUVEC)	75% reduction in TRPM4 mRNA and ~50% reduction in TRPM4 protein	TRPM4 is involved in endothelial injury induced by arsenic trioxide	[145]
Leukemia cell lines with the MLL gene rearrangement	75% reduction in TRPM4 mRNA and ~50% reduction in TRPM4 protein	TRPM4 may be involved in the pathogenesis of MLL-rearranged leukemia	[146]
Permanent middle cerebral artery of rat	Prevented the expression of TRPM4	TRPM4 upregulation contributes to cerebral damage in acute phase of stroke	[141]
Bilateral common carotid arteries occlusion rat models	Prevented the expression of TRPM4	TRPM4 mediates cognitive deficits and LTP impairment and reduces the expression of synaptic proteins	[142]
HUVEC	At least 90% reduction in TRPM4 mRNA and protein	TRPM4 is involved in lipopolysaccharide-induced endothelial cell death	[140]
Jurkat cells	Some reduction in TRPM4 mRNA and protein	TRPM4-mediated depolarization modulates Ca^2+^ oscillations	[147]

**Table 2:** Studies with TRPM4 dominant-negative variants.

Type of Variant	Studied Preparation	Results	Conclusion	Reference
Single amino acid modification (D984A)	HEK cells	Nonconducting TRPM4 channels	Information about the selectivity filter of TRPM4	[148]
D984A variant	Colorectal cancer cell line HCT116	Complete inhibition of the current without the reduction in TRPM4 protein expression	Ion conduction of TRPM4 plays a versatile role in cancer cell proliferation, cell cycle, and invasion	[24]
D984A variant	Human prostate cancer cells line	Nonconducting TRPM4 channels	TRPM4 is involved in cancer hallmark functions (cell viability, proliferation, migration, and cell cycle shift)	[132]
Deletion of first 177 amino acids in the N-terminus (ΔN-TRPM4)	Jurkat cells	Hardly conducts any current	TRPM4-mediated depolarization modulates Ca^2+^ oscillations	[147]
ΔN-TRPM4	HUVEC	Suppression of TRPM4 activity	TRPM4 contributes to lipopolysaccharide-induced endothelial cell death	[140]
ΔN-TRPM4	Rat insulinoma cell line INS-1	Reduced TRPM4-mediated current	TRPM4 is involved in glucose- or arginine-vasopressin-induced insulin secretion	[25]
ΔN-TRPM4	Rat insulinoma cell line INS-1	Reduced TRPM4-mediated current	TRPM4 contributes to calcium signals and insulin secretion	[149]

## Data Availability

Data sharing not applicable.

## Abbreviations

AP	action potential
ATP	adenosine triphosphate
BTP2	3,5-bis(trifluoromethyl)pyrazole derivative
CBA	4-chloro-2-[[2-(2-chlorophenoxy)acetyl]amino]benzoic acid
CFTR	cystic fibrosis transmembrane conductance regulator
CHO	Chinese hamster ovary
DCDPC	3′,5-dichlorodiphenylamine-2-carboxylic acid
DPC	diphenylamine-2-carboxylic acid
DV	decavanadate
EC_50_	half effective activator concentration
FFA	flufenamic acid
HEK	human embryonic kidney
HUVEC	human umbilical vein endothelial cells
IC_50_	half inhibitory concentration
IP3	inositol 1,4,5-trisphosphate
I(NSC_Ca_)	Ca^2+^-activated nonspecific cationic current
K_ATP_	adenosine triphosphate-dependent K^+^
KCax.x	Ca^2+^-activated K^+^ channels type x.x
Kirx.x	inward rectifier K^+^ channel type x.x
KD	knock-down
KO	knock-out
LBA	4-chloro-2-(2-(4-chloro-2-methylphenoxy)propanamido) benzoic acid
LNCaP	lymph node carcinoma of the prostate
NBA	4-chloro-2-(1-naphthyloxyacetamido)benzoic acid
NMDA	*N*-methyl-d-aspartic acid
NO	nitric oxide
NPPB	5-nitro-2-(3-phenylpropylamino)benzoic acid
NSC_Ca_	Ca^2+^-activated nonspecific cationic channel
PIP2	phosphatidylinositol 4,5-bisphosphate
PKC	protein kinase C
PLC	phospholipase C
PMA	phorbol 12-myristate 13-acetate
tPA	tissue plasminogen activator
siRNA	small interfering RNA
SUR1	sulfonylurea receptor 1
TRP	transient receptor potential
TRPA	transient receptor potential ankyrin
TRPC	transient receptor potential canonical
TRPM	transient receptor potential melastatin
TRPML	transient receptor potential mucolipin
TRPP	transient receptor potential polycystin
TRPV	transient receptor potential vanilloid
